# Aortitis as a Rare Cause of Aortic Aneurysm and Valve Regurgitation: Is Repair Precluded?

**DOI:** 10.1155/2018/5757081

**Published:** 2018-02-13

**Authors:** Carlos Porras, Gemma Sanchez-Espin, Miguel Such, Jesús Sánchez-Ramos, Alicia Bautista-Pavés, Pilar Martín-de la Fuente, Josefa Ruiz, Norberto Ortego-Centeno, Isabel Rodríguez-Bailón, José María Melero

**Affiliations:** ^1^Cardiac Surgery, Hospital Universitario Virgen de la Victoria, Málaga, Spain; ^2^Cardiac Surgery, Hospital Xanit Internacional, Málaga, Spain; ^3^Cardiology, Complejo Hospitalario Universitario de Granada, Granada, Spain; ^4^Internal Medicine, Hospital Universitario Virgen de la Victoria, Málaga, Spain; ^5^Internal Medicine, Complejo Hospitalario Universitario de Granada, Granada, Spain; ^6^Cardiology, Hospital Universitario Virgen de la Victoria, Málaga, Spain

## Abstract

Aortitis is an infrequent cause of aortic root dilatation and aortic valve regurgitation. Valve-sparing procedures have been proposed, but there is not clear evidence of which is the treatment of choice. We report the case of a 38-year-old pregnant lady with a diagnosis of idiopathic aortitis associated with aortic root aneurysm and severe aortic valve regurgitation.

## 1. Introduction

Aortitis is an infrequent cause of aortic root dilatation and aortic valve regurgitation, having been described several infectious and noninfectious etiologies for this yet not well-known entity [1]. The approach to the “valvular problem” is controversial, having been reported increased rates of paravalvular leaks with prosthetic replacement [2]. Valve-sparing procedures have also been proposed [3], but there is not clear evidence of which is the treatment of choice.

## 2. Case Presentation

We report the case of a 38-year-old pregnant lady with a diagnosis of idiopathic aortitis associated with aortic root aneurysm and severe aortic valve regurgitation.

The patient had a history of cardiac murmur, having had an echocardiogram performed two years before the episode showing a mild mitral regurgitation with a normal aortic valve and aorta. She was asymptomatic until the 4th month of pregnancy when she began with tachycardia and exertional dyspnea which rapidly evolved to rest dyspnea; she was admitted to the hospital, and an echocardiographic study was performed, showing a very severe aortic regurgitation and a 5.5 cm aneurysm of the root and ascending aorta; the left ventricle was markedly dilated with a borderline ventricular fraction. No computed tomography scan was done to avoid potential damage to the fetus.

She was transferred to our unit for urgent surgery for a planned valve-preserving surgery.

She was operated on under general anesthesia and median sternotomy. The aortic arch and the right atrium were cannulated in a standard fashion, and cardiopulmonary bypass (CPB) was established under normothermia. CPB flows were kept high maintaining a mean arterial pressure above 70 mmHg, and the use of vasoconstrictors was avoided [4, 5].

The aorta was clamped and opened showing a marked thickening of its wall. The root was dissected, and the coronary ostial buttons trimmed; the tissues around the root had heavy adherences indicating an inflammatory process. Geometric height of the leaflets was measured with a ruler; it was 15 for the noncoronary leaflet, 14 for the right, and 14 for the left. A valve-sparing remodeling procedure was done using a dacron straight graft of 24 mm. The technique of the remodeling procedure followed the recommendations of the Homburg group [6]. The post procedure intraoperative echocardiogram showed a residual mild regurgitation of the valve, which was considered acceptable. The jet was central, in its origin and direction, and the effective height of the repaired cusps was 8 mm.

The fetal status was assessed before surgery and after the completion of the procedure by transabdominal echography, showing an alive fetus.

The postoperative course of the patient was uneventful. She was discharged on metoprolol and iron. A transthoracic echocardiogram was performed before discharge showing a mild regurgitation of the aortic valve with a marked unloading of the left ventricle.

She continued her pregnancy, and a healthy girl was born with a vaginal delivery.

She was investigated thoroughly, and all known causes of aortitis or genetic disorders were discarded. No other signs or symptoms of the disease were found. She was diagnosed with idiopathic aortitis at that moment. Anyhow, she was started with immunosuppressant treatment with corticoids.

She underwent clinical and serial echocardiographic follow-up. The 6-month echo showed a progression of the regurgitation which was moderate at that time, and in the 1-year echo, it became severe. As she continued to be asymptomatic, a narrower follow-up was decided, but 3 months later, the left ventricle had dilated markedly, and redo surgery was indicated.

Redo surgery was performed through a resternotomy with central cannulation. The Dacron graft was opened through a transverse incision, the aortic valve was measured and resected, and a 23 mm mechanical prosthesis was inserted in a supra-annular position with pledgeted sutures. The leaflets were markedly retracted at that time, with a geometric height of less than 4 mm. The Dacron graft was then sutured, and the patient was weaned from CPB and transferred to the Intensive Care Unit for an uneventful recovery.

Two years later, a new-onset supra-aortic vessel narrowing has been detected, and a diagnosis of Takayasu disease has been made.

## 3. Discussion

Aortitis is a rare cause of aortic aneurysms and aortic valve regurgitation.

Aortitis of the ascending aorta is a rare condition that can result in aneurysm and valve dysfunction. Although valve-preserving surgery has been successfully accomplished by others [3], in our opinion, these patients might probably be best served by valve and root replacement.

In our patient, the aorta wall was markedly thickened and the geometric height of the leaflets was low which should have led us to replace the valve instead of performing a valve-preserving surgery as formerly showed by the evolution of the patient. Geometric height is a major prognostic factor in valve repair with heights under 16 mm in tricuspid valves or 19 mm in bicuspid valves considered as restricted, being advisable to replace the valve in those instances [7].

The mechanism because of which the valve was regurgitant was not only the dilation of the aorta but also, and probably most importantly, the retraction of the tissue of the leaflets which was responsible for the recurrence of the aortic insufficiency.

Considering the pregnancy of the patient, we tried to avoid a prosthetic valve substitute and preserved the valve despite its low geometric height. She could continue her pregnancy until term without any damage to the newborn. Anyway, given the evolution of the case, probably she would have been better served with a biological root replacement.

Follow-up of repaired patients is mandatory, with 6-month and yearly echocardiographic assessment highly advisable in our opinion, to discard recurrence of the regurgitation, which happened in our patient, who despite being asymptomatic had progressive dilatation of the left ventricle.

## 4. Conclusions

Aortic valve repair is probably not the best option for patients with aortitis.

Close follow-up of these patients is mandatory.
